# Pediatric Intensive Care: Immunomodulation With Activated Protein C *ex vivo*

**DOI:** 10.3389/fped.2019.00386

**Published:** 2019-09-27

**Authors:** Hassan O. Eliwan, William R. G. Watson, Irene Regan, Brian Philbin, Fiona M. O'Hare, Tammy Strickland, Amanda O'Neill, Michelle O'Rourke, Alfonso Blanco, Martina Healy, Beatrice Nolan, Owen Smith, Eleanor J. Molloy

**Affiliations:** ^1^Neonatology, National Maternity Hospital, Dublin, Ireland; ^2^National Children's Research Centre, Dublin, Ireland; ^3^Paediatrics, Royal College of Surgeons in Ireland, Dublin, Ireland; ^4^University College Dublin School of Medicine and Medical Sciences, Conway Institute of Biomolecular and Biomedical Science, University College Dublin, Dublin, Ireland; ^5^Haematology, Our Lady's Children's Hospital, Dublin, Ireland; ^6^Paediatrics, Trinity College, The University of Dublin, Dublin, Ireland; ^7^Critical Care, Our Lady's Children's Hospital, Dublin, Ireland; ^8^Neonatology, Our Lady's Children's Hospital, Dublin, Ireland; ^9^Paediatrics, Coombe Women's and Infant's University Hospital, Dublin, Ireland

**Keywords:** PICU, Sepsis, APC, CD11b, TLR4, ROI, LPS

## Abstract

**Objective:** Sepsis is major cause of morbidity and mortality in the Pediatric Intensive Care Unit (PICU). PICU patients may develop transient immune deficiency during sepsis. Activated Protein C (APC) has significant anti-inflammatory and cytoprotective effects. Clinical trials of APC in adult sepsis initially showed improved outcome but recent trials showed no benefit in adults or children. We aimed to assess the effects of APC treatment on innate immune responses in children.

**Design and Subjects:** We compared neutrophil and monocyte responses to lipopolysaccharide (LPS) with and without APC treatment in PICU patients at the time of evaluation for sepsis compared with healthy adults and age-matched pediatric controls. We used flow cytometry to examine cell activation (CD11b expression), function [intracellular reactive oxygen intermediate (ROI) release] and LPS recognition [Toll like Receptor 4 (TLR4) expression].

**Results:** PICU patients had significantly decreased protein c levels and LPS responses compared with adult and pediatric controls for all parameters. APC reduced LPS-induced neutrophil PICU TLR4 and adult ROI (*p* < 0.05). PICU non-survivors had increased LPS induced neutrophil and monocyte ROI production vs. survivors which was significantly reduced by APC.

**Conclusion:** PICU patients demonstrate significantly reduced endotoxin reactivity which may predispose them to sepsis and alter effective antibacterial responses. APC reduces LPS-induced ROI production in adults and may have a role in treating severely compromised PICU patients especially given that newer APC forms are associated with decreased bleeding risk and enhanced anti-inflammatory effects.

## Introduction

Sepsis is the 2nd leading cause of death in children and the 4th leading cause of death in infants. The incidence of sepsis in USA is 0.56/1,000 children with an overall mortality of 10.6% and an overall cost of almost 2 billion dollars per annum (1). Sepsis is characterized by a systemic inflammatory response to infection and may result in tissue damage, multi-organ dysfunction (MOD), and death. Persistent activation of leukocytes may contribute to this process while excessive leukocyte elimination may contribute to immunoparesis, bacterial overgrowth, and death. Establishing a balance between over-activation of leukocytes and excessive leukocyte elimination reduces tissue damage and mortality (2).

Septic adult patients often demonstrate defects in cell surface antigen expression and reactive oxygen intermediate (ROI) production, dysregulation of cytokine release, and enhanced apoptosis (3). We were interested in studying immune cell LPS recognition, activation and adhesion in Pediatric Intensive Care Unit (PICU) patients.

Toll-Like Receptors (TLRs) are a group of vital transmembrane receptors that initiate innate immune responses to an array of micro-organisms. TLR4 is the primary receptor that recognizes lipopolysaccharide (LPS) and mediates nuclear factor-kappa B (NF-kB) activation and proinflammatory cytokine synthesis (4). Baseline monocyte and neutrophil TLR4 expression is increased in adult sepsis (5).

CD11b is a subunit of the β2 integrin adhesion molecule and is expressed on myelocytes and more mature granulocytes (6). Upon binding bacterial LPS, neutrophil and monocyte expression of CD11b increases (7). Weirich et al. demonstrated increased neutrophil CD11b expression in infants with infection (6). Similarly, adults with sepsis and organ dysfunction show increased monocyte CD11b expression (8).

Reactive Oxygen Intermediate (ROI) production is essential for intracellular killing of invading microorganisms following phagocytosis by neutrophils. Intracellular ROI generation is upregulated both in neutrophils and monocytes in adults with sepsis (9). Neutrophil respiratory burst is increased in neonates showing signs of infection with *Escherichia coli* (10).

In the era of increasing antibiotic resistance, the development of adjunctive therapies for sepsis is crucial. Protein C is an endogenous vitamin K dependent glycoprotein that circulates in plasma as an inactive zymogen. Activated Protein C (APC) plays a role in coagulation and regulation of inflammation (11). In the PROWESS (Recombinant human Protein C World Evaluation in Severe Sepsis) study, the administration of APC reduced mortality in adult patients with severe sepsis and high risk of death (12). However, subsequent studies showed no benefit and APC (Xigris) was withdrawn from the market by Eli Lilly. A phase 1b open-label study found that the pharmacokinetics and pharmacodynamics of APC are similar in children and adults. However, the RESOLVE (Researching severe Sepsis and Organ dysfunction in children: a global perspective) study investigated the efficiency and safety of APC in children and concluded that it had no effect in children with sepsis (13). Yet recently modified versions of APC have been developed as immunomodulators in sepsis and have demonstrated less haemorrhagic potential and greater anti-inflammatory properties.

We hypothesized that APC diminishes neutrophil and monocyte activation and therefore reduces tissue damage in both adult and neonatal sepsis. In this study, we aimed to investigate neutrophil and monocyte CD11b/TLR4 expression and ROI production following LPS stimulation in pediatric sepsis patients *ex vivo* and to investigate the effect of APC treatment on these responses.

## Materials and Methods

### Reagents

Lipopolysaccharide (LPS) *E. coli* serotype 0111:B4, Phorbol 12-Myristate 13-Acetate (PMA), Dihydrorhodamine 123 (DHR), Fetal Bovine serum (heat inactivated), and phosphate buffered saline (PBS) were purchased from Sigma Aldrich Ltd. (www.sigmaaldrich.com). Dulbecco's modified Eagle's medium (DMEM) was purchased from Bioscience Ltd. (www.biosciences.ie). Penicillin streptomycin solution and l-glutamine serum (FCS) were purchased from GIBCO Life Technologies (www.invitrogen.com). *Drotrecoginalfa* (Xigris: Activated Protein C [APC]) was purchased from Eli Lilly (www.lilly.ie). CD11b was purchased from eBioscience (www.ebioscience.com). FACS lysing solution and Toll like Receptor (TLR4) were purchased from BD Bioscience (www.bdbiosciences.com) and all remaining chemicals were purchased from Sigma Aldrich company Ltd (Dorset UK) unless otherwise stated.

### Patient Population

This study was approved by the Institutional Research Ethics Committee and fully informed written consent was obtained from all participants. The PICU in Our Lady's Children's Hospital Crumlin (OLCH) is the largest in Ireland and has 21 bedded Intensive Care/HDU. The adult control group consisted of healthy adult volunteers from the laboratory and hospital. Pediatric controls included children admitted to the surgical day ward for elective minor surgical procedures with no significant medical history. Samples were taken in theater during cannula insertion by the anesthetic team.

The PICU group included children admitted to the PICU for different medical and surgical conditions who underwent a septic workup for clinical suspicion of sepsis including fever, clinical deterioration, or increased inflammatory markers. Patients with neutropenic sepsis, malignancy, and cardiac bypass surgery in the preceding week, primary immune deficiency or who were older than 12 years were excluded from this study. The septic workup included a full blood count (FBC), C-reactive protein (CRP), Blood, sputum, and urine cultures as well as a coagulation screen (PT, APTT, Fibrinogen), Protein C levels and lumbar puncture if clinically indicated. Detailed information was taken from patient records including multiple organ outcome data to allow scoring of the degree of organ dysfunction (14).

### Sample Processing

Whole blood samples were kept in ice and processed within 90 min. Whole blood was incubated for 1 h at 37°C with stimulatory agent LPS 20 ng/mL to mimic an inflammatory response *in vitro*. In addition APC 200 ng/mL was added ±LPS following a dose response study (results not shown) and in accordance with the *in vitro* studies of Galley et al. (15).

### Protein C Activity (Chromogenic)

The Protein C level in patient plasma is measured in two stages: Incubation of the plasma with Protein C Activator; and quantification of Activated Protein C with a synthetic chromogenic substrate. Paranitroaniline released is monitored kinetically at 405 nm and is directly proportional to the Protein C level in the test sample. The HemosIL Protein C Kit used contains lyophilized substrate, Protein C Activator, and a saline diluent. Blood was collected (vol. 9) in 0.109 M (i.e., 3.2%) trisodium citrate anticoagulant bottles (Vol. 1) and samples were centrifuged at 4,000 rpm (2,500 rcf/g) for 10 min. The testing was completed within 4 h of sample collection. The sample was inverted a number of times or vortexed to ensure adequate mixing. Mixing is critical before testing, as precipitation of certain proteins may occur with freezing. Samples should be tested immediately. Any samples that were clotted or appeared haemolyzed were rejected. To ensure internal quality control, two different controls (Normal Control and HemosIL Low Abnormal) were included at the start of each working day and subsequently every 4 h throughout the day.

### Quantification of Cell Surface Antigen Expression

The expression of CD11b and TLR-4 antigens on the surface of neutrophils and monocytes was measured by flow cytometry. Whole blood (50 μL) was treated with 5 μL of PE-CD11b and 2.5 μL of anti-human TLR4 antibodies and was then left at 4°C for 20 min. FACS was added and incubated for 10 min at room temperature. The sample was centrifuged at 3,000 rpm for 5 min at 4°C. The pellet was resuspended twice with DMEM 500 μL and stored on ice before analysis by flow cytometry. The fluorescence intensity is denoted by mean channel fluorescence, which is the average intensity of fluorescence emitted by all cells chosen for measurement and is comparable to the relative number of receptors present on the surface of each cell. The flow cytometer used was a BD Accuri C6 and a minimum of 5,000 events were collected and analyzed with a CFlow Plus software. All measurements were performed under the same instrument settings (16–20). Scatter profiles we used to delineate leukocyte populations using forward scatter and side scatter. Whole blood was used and neutrophil and monocyte populations were gated and confirmed by cell sorting in Flow Cytometer gates. A consultant hematologist and histologist then examined the sorted cells which had been fixed on a slide, stained, and analyzed under a microscope (16).

### Respiratory Burst Activity

Generation of ROI was evaluated by flow cytometry using the technique of Smith and Wiedemann (17). Whole blood (50 μL) was incubated with DHR 123 (50 μL) and PBS (450 μL) at 37°C for 10 min. Cells were stimulated with 1 μL (16 μM) phorbol 12-myrisate 13-acetate (PMA) for 20 min at 37°C. The reaction was then halted by placing samples on ice. Neutrophil and monocyte fluorescence intensity was assessed by flow cytometry and expressed as Ln mean channel fluorescence (LnMCF). DHR 123 has been shown to detect intracellular H_2_O_2_ and OH radical production (21).

### Statistical Methods

PASW statistical package version 18 (IBM Corp, Chicago, IL, USA; www.ibm.com/SPSS Statistics) was used to statistically analyse experimental and clinical data. For comparing conditions (Control/LPS/LPS+APC) within each group (Adults/Pediatric/PICU) matched paired *t*-tests were used. For comparing across groups one -way ANOVA with Tukey *post-hoc* comparison method was used and a *p* < 0.05 was considered significant.

## Results

### Patient Demographics

There were 15 adult controls, 25 pediatric controls and 15 PICU patients enrolled in this study. The adult controls had a median age of 26 years (22–38 years) with nine males and six females. The pediatric controls had a median age 21 months (4 months−12 years) including 14 males and 11 females. The PICU group had a median age 6 months (1 week−11 years) and there were seven males and eight females. The most common diagnosis in the PICU group was congenital heart disease (Table 1). Five children died during their PICU stay and all children enrolled were on antibiotics at the time of sampling. Cultures from blood, sputum, central line, or urine were positive in 12 out of 14 patients (15 samples) and one blood culture was positive (Table 1). Therefore, only one child had culture-positive sepsis although nine children had abnormal CRP levels (range 11–320 mg/l) and were managed as culture-negative sepsis. The Multiple organ dysfunction scores (MODS) were as follows: 4 organs (*n* = 1); 3 organs (*n* = 6); 2 organs (*n* = 5); 1 organ (*n* = 2); 0 organs (*n* = 0) (14).

**Table 1:** Clinical and laboratory characteristics of PICU patients.

**Patient**	**Age range**	**Diagnosis**	**Death**	**Co-morbidity**	**Microbial growth**	**Culture site**	**CRP**
1	12 y	T21, ASD,VSD, Pulmonary stenosis	N	LRTI	*Ps. aeruginosa*	Sputum	4
2	12 m	T21, AVSD	N	CCF, LRTI	*E. coli*	Sputum	4
3	12 y	Aspiration pneumonia	N	Respiratory failure	*H. Influenzae* and *Strep. pneumoniae*	Sputum	230
4	12 m	Scimitar syndrome	Y	Seizures, RSV bronchiolitis, LRTI	No growth	NA	70
5	12 y	Spinal fusion for idiopathic scoliosis	N	Left pulmonary collapse	No growth	NA	152
6	30 d	HLHS	N	Cardiac tamponade, coagulopathy	No growth	NA	20
7	12 m	CDH	Y	PPHN, PDA	*Coag Neg staph*	Central line	4
8	12 y	Pneumonia with pleural effusion	N	Empyema	*Stenotrophomonas maltophilia*	Sputum	8
9	30 d	Aortic stenosis & endocardial fibroelastosis	N	Seizures, RSV brochiolitis	*Kl. oxytoca*	Central line	103
10	12 m	Perforated NEC	N	Preterm, grade II IVH	*Coag Neg staph*	Central line	40
11	30 d	Duodenal atresia	Y	IVH	*E. coli*	Blood	67
12	12 m	TOF	N	Chylothorax	*Ps. aeruginosa*	Central line	4
13	30 d	Perforated NEC	N	SVT	*Enterococcus* spp. and *Staph coag neg*	Central line	118
14	30 d	HLHS	Y	Renal Failure	*Candida glabrata*	Urine	32

*T21, Trisomy 21; ASD, atrial septal defect; VSD, ventricular septal defect; LRTI, lower respiratory tract infection; AVSD, atrial ventricular septal defect; CCF, congestive cardiac failure; RSV, respiratory syncitial virus; HLHS; hypoplastic left heart syndrome; CDH, congenital diaphragmatic hernia; PPHN, persistent pulmonary hypertension; PDA, patent ductus arteriosus; NEC, necrotizing enterocolitis; PT, preterm; TOF, Tetralogy of Fallot; SVT, supraventricular tachycardia; Ps. aeruginosa, Psedomonas aeruginosa; Coag Neg staph, Coagulase negative staphylococcus; H.influenzae; Haemophilus influenza; Strep. Pneumoniae, Streptococcus pneumonia; Kl. oxytoca, Klebsiella oxytoca*.

### Protein C Levels

Adult controls had Protein C levels within or above the normal range with the median (range) 1.29 (0.94–2.29) mg/L. All pediatric controls had normal Protein C levels: 0.7 (0.47–1.04) mg/L. Protein C levels were available in seven PICU patients: 0.52 (<0.01–0.99) mg/L. Only one PICU patient had a low Protein C level (<0.01) and this patient died.

### Reactive Oxygen Intermediates

**Figure 1:**
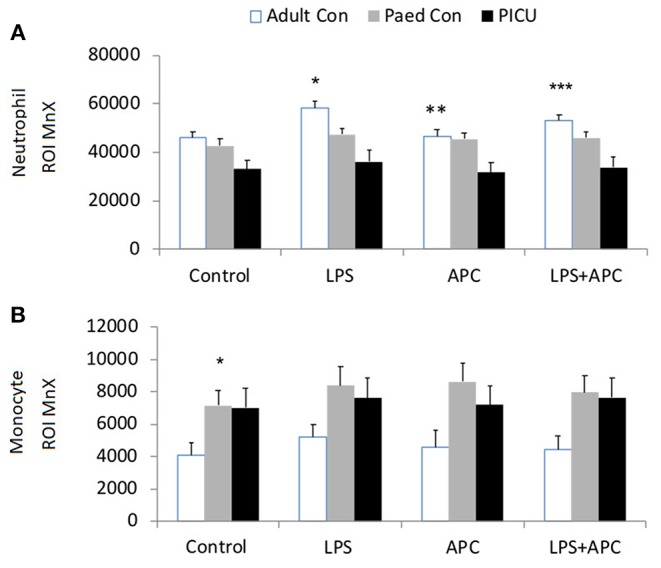
Neutrophil and monocyte LPS responses and APC modulation: Whole blood from healthy adult controls (*n* = 15), pediatric controls (*p* = 15), and PICU patients (*n* = 15) was incubated with LPS + APC for 1 h. Then stimulated with DHR and PMA. Results were expressed as the Ln mean channel fluorescence + SEM. **(A)** Neutrophil ROI: *p < 0.05 vs. pediatric controls and PICU patients; **p < 0.05 vs. adult controls; ***p < 0.05 vs. adult LPS response. **(B)** Monocyte ROI: *p < 0.05 vs. adults controls.

A significantly increased ROI release was noted in adult control neutrophils in response to LPS. LPS also induced an increased ROI release from pediatric control PMNs although not to the same degree as adults. In contrast, PICU patients were LPS hyporesponsive. There was a significant decrease in LPS stimulated ROI release from adults following APC (*p* = 0.01). However, there were no changes in pediatric controls or PICU patients' ROI responses to APC (Figure 1A).

Monocytes produce ~80% less ROIs than neutrophils. Monocytes showed the same trends as seen in neutrophils with increased monocyte ROI production in adult and pediatric controls in response to LPS compared to PICU patients (*p* = 0.015 and *p* = 0.049, respectively; Figure 1B).

### Neutrophil and Monocyte Cell Surface Receptor Expression

**Figure 2:**
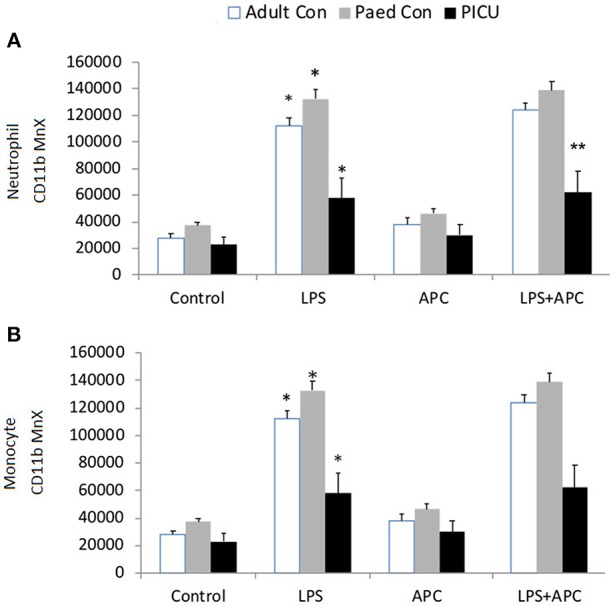
Neutrophil and monocyte CD11b expression: Whole blood from healthy adult controls (*n* = 15), pediatric controls (*p* = 15) and PICU patients (*n* = 15) was incubated with LPS + APC for 1 h. Neutrophils and monocytes were assessed for CD11b expression using a PE-labeled mAb and mean channel fluorescence analyzed using flow cytometry. The neutrophil and monocyte populations were selected based on their scatter profile: forward scatter and side scatter **(A)** Neutrophil CD11b expression: *p < 0.05 vs. respective controls; **p < 0.05 vs. PICU patients. **(B)** Monocyte CD11b expression: *p < 0.05 vs. respective controls.

There was a significant increase in neutrophil CD11b in all groups in response to LPS. There was increased LPS responsiveness in adult and pediatric controls compared to PICU patients. APC had no effect in reducing LPS stimulated neutrophil CD11b in any group (Figure 2A). Similar CD11b responses were seen in monocytes and there was increased LPS stimulated monocyte CD11b in all groups. However, monocytes from PICU patients were significantly LPS hyporesponsive compared with adults and pediatric controls (*p* = 0.012 and *p* = 0.001, respectively; Figure 2B).

**Figure 3:**
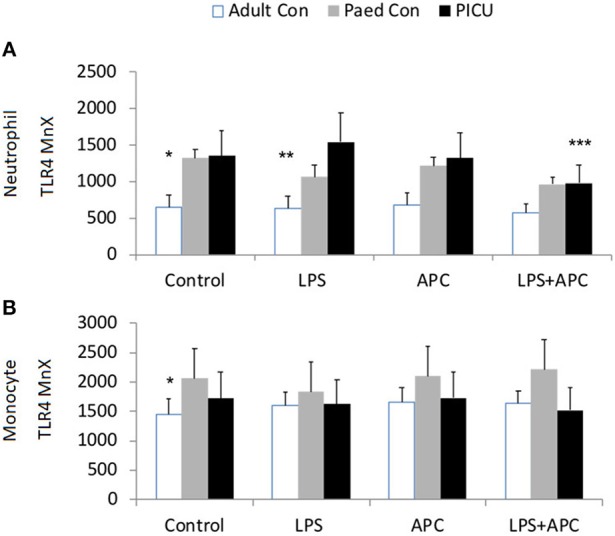
Neutrophil and monocyte TLR4 responses and APC modulation: Whole blood from healthy adult controls (*n* = 15), pediatric controls (*p* = 15), and PICU patients (*n* = 15) was incubated with LPS + APC for 1 h. Neutrophils and monocytes were then labeled with Alex Fluor 647 TLR4 mAb and mean channel fluorescence was analyzed using flow cytometry. The neutrophil and monocyte populations were selected via flow cytometry based on their scatter profile: forward scatter and side scatter. **(A)** Neutrophil TLR4 expression: *p < 0.05 vs. PICU control; **p < 0.05 vs. PICU LPS response; ***p < 0.05 vs. PICU LPS response. **(B)** Monocyte TLR4 expression: *p < 0.05 vs. PICU controls.

Adults and pediatric controls did not show increased neutrophil TLR4 expression in response to LPS. Increased neutrophil expression of TLR4 was noted in response to LPS in PICU patients (*p* = 0.02). This was significantly reduced by APC (*p* = 0.005; Figure 3A). There was no increase in monocyte TLR4 expression in response to LPS in any group (Figure 3B).

### Comparison of PICU Survivors vs. Non-survivors

**Figure 4:**
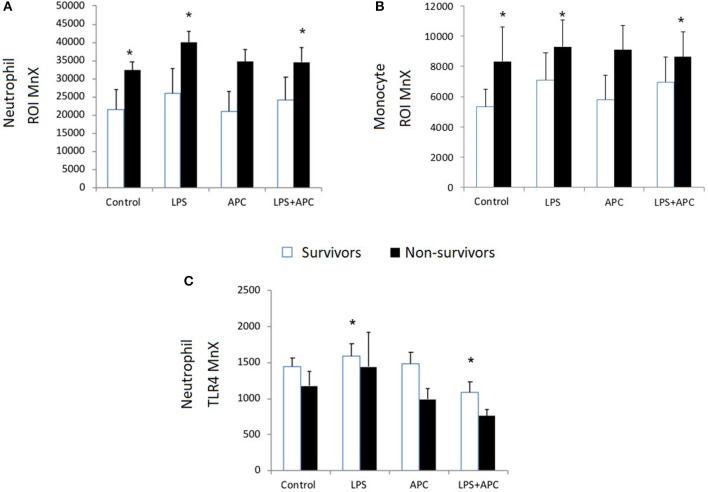
Neutrophil and Monocyte ROI and Neutrophil TLR4 expression in survivors and non-survivors in PICU: Whole blood was incubated with LPS ± APC for 1 h and then stimulated with DHR and PMA and evaluated by flow cytometry. Results were expressed as the Ln mean channel fluorescence + SEM. **(A)** Neutrophil ROI generation: *p < 0.05 vs. survivor controls. **(B)** Monocyte ROI generation: *p < 0.05 vs. survivor controls. **(C)** Neutrophil TLR4 expression: Whole blood was incubated with LPS ± APC for 1 h. Neutrophils were labeled with Alex Fluor 647 TLR4mAb and mean channel fluorescence (MCF) analyzed using flow cytometry. *p < 0.05 vs. non-survivor controls.

Baseline neutrophil and monocyte ROI expression was higher in PICU non-survivors compared to survivors. Non-survivors had significant increased neutrophil and monocyte ROI production in response to LPS in contrast to survivors (*p* = 0.001). Non-survivors LPS-induced ROI production was significantly reduced by APC (*p* = 0.034; Figures 4A,B). There was no significant difference in CD11b expression at baseline in survivors and non-survivors. Neutrophil and monocyte CD11b was significantly increased in both groups with LPS and was not altered by APC. There was no significant difference at baseline in neutrophil and monocyte TLR4 expression in non-survivors vs. survivors. Similarly, there was no significant difference between neutrophil and monocyte TLR4 expression in response to LPS in these groups (Figure 4C).

## Discussion

PICU patients were hyporesponsive to LPS with decreased neutrophil and monocyte CD11b and ROI expression. Repetitive stimulation of monocytes with LPS leads to decreased responsiveness termed “endotoxin tolerance” (22). Relative endotoxin hyporesponsiveness in PICU patients may relate to endogenous endotoxin stimulation and exhaustion of neutrophils and monocytes *in vivo*. Neutrophils and monocytes from adults with sepsis have reduced activation and production of pro inflammatory cytokines (IL-1α, IL-6, TNF-α) in response to LPS (23, 24). Overwhelming sepsis is associated with a significant reduction of neutrophil chemotaxis (25) and adherence (26). This immune hyporeactivity state (immmunoparesis) during sepsis may explain the persistence of infection, late nosocomial infection, and death (27). In the PICU, immunoparalysis is associated with a higher risk of nosocomial infection and mortality (28).

Neutrophil CD11b expression in response to LPS increases in human whole blood in a dose-dependent manner (29). Neutrophil and monocyte CD11b expression is significantly increased in infected compared to non-infected and healthy neonates (30, 31). We have shown significantly increased neutrophil and monocyte CD11b expression in pediatric and adults controls vs. PICU patients. Recently APC has been shown to inhibit NETosis in neutrophils via a Mac-1 (CD11b) dependent mechanism (15, 32).

We showed increased neutrophil ROI release in response to LPS in both adult and pediatric controls compared to PICU patients. Increased ROI generation is often linked with tissue damage and is increased in multiorgan dysfunction (MOD) associated with the systemic inflammatory response syndrome (SIRS) in adult sepsis (33, 34). In addition, increased monocyte and neutrophil ROIs are associated with severe sepsis in adults (9). However, in this study LPS-induced ROI responses were suppressed in PICU patients, which may be associated with immunoparesis.

Neutrophil TLR4 expression in PICU patients was increased vs. pediatric and adult controls in response to LPS. APC significantly reduced neutrophil LPS-induced TLR4 expression in PICU patients only. Previous studies have concentrated only on baseline unstimulated innate immune cell function with APC. Viemann et al. found that baseline unstimulated neutrophil TLR4 expression was significantly higher in septic children vs. healthy controls (35). Previous studies have shown that baseline monocyte expression of TLR2 and TLR4 in patients with sepsis in intensive care is significantly up-regulated compared with healthy controls (5). Additionally, it has been demonstrated that baseline monocyte TLR4 expression is increased in septic compared to healthy neonates (36) and that isolated neutrophils and monocytes from adults with sepsis have higher mean fluorescence for TLR4 expression than cells from controls (37).

In this study, APC decreased the LPS-induced neutrophil ROI response in adults. However, this response was not seen in PICU patients as they were deemed LPS hyporesponsive and already demonstrated minimal ROI production. We found that despite using isolated neutrophils and not whole blood, there was no significant reduction in LPS-induced ROI with APC. These findings were supported by other groups and this response may be attributable to a plasma factor (38). APC decreased adult ROI LPS responses but this was not found in pediatric controls or PICU patients. The differences in adult and pediatric immune responses to APC may partially explain the differences in outcome of the adult APC sepsis trial vs. the pediatric groups in the PROWESS and RESOLVE trials. PICU non-survivors had increased neutrophil LPS responses which decreased with APC suggesting a possible role for APC or its variants in a subgroup of children with sepsis. However, in view of the potential for hemorrhage associated with APC treatment, the study of newer forms of APC with decreased haemorrhagic potential and enhanced anti-inflammatory effect is essential. A mutant APC generated with three alanine mutations (3K3A-APC) has been created and demonstrates potent neuroprotective properties and reduced anticoagulant activity. The creation of mutant APC forms with less anticoagulant activity may increase the utility of APC in sepsis especially in populations vulnerable to hemorrhage (39). Further studies (including protein engineering studies) aiming to minimize side-effects while preserving therapeutic and cytoprotective effects of APC are ongoing (40).

Eli Lilly and Company announced a worldwide voluntary market withdrawal of Xigris [Drotrecogin alfa (activated)] in 2011 following the PROWESS-SHOCK study which failed to show a survival benefit for patients with severe sepsis and septic shock (41). We have demonstrated that patients who undergo a sepsis evaluation in PICU have homogenous immune responses to endotoxin despite their heterogeneous diagnoses. Understanding endotoxin tolerance in PICU patients may assist in creating future therapies for pediatric sepsis. In addition, further understanding the sepsis-protective activities of variants of APC will be essential to explore how to administer APC in a clinical setting.

## Data Availability Statement

All datasets generated for this study are included in the manuscript/supplementary files.

## Ethics Statement

This prospective study was carried out in accordance with the recommendations of the National Children's Research Centre guidelines. Fully informed written parental consent was obtained from all pediatric participants and fully informed written consent was likewise obtained from all adult controls in compliance with the Declaration of Helsinki. The protocol was approved by the Institutional Research Ethics Committee in Our Lady's Children's Hospital Crumlin (OLCHC) Dublin.

## Author Contributions

HE, TS, and EM composed and edited the manuscript. HE and TS analyzed data and put together the included data figures and tables. WW, FO'H, AO'N, and AB assisted in sample preparation, laboratory technique troubleshooting, data analysis, and advised on and edited the manuscript. IR, BP, MO'R, MH, BN, and OS assisted HE in patient recruitment, data collection, sample collection, and advised on and edited the manuscript.

### Conflict of Interest

The authors declare that the research was conducted in the absence of any commercial or financial relationships that could be construed as a potential conflict of interest.
